# Clinical Proteomics Profiling for Biomarker Identification Among Patients Suffering With Indian Post Kala Azar Dermal Leishmaniasis

**DOI:** 10.3389/fcimb.2020.00251

**Published:** 2020-05-27

**Authors:** Priyank Jaiswal, Manab Ghosh, Goutam Patra, Bibhuti Saha, Sumi Mukhopadhyay

**Affiliations:** ^1^Department of Laboratory Medicine, School of Tropical Medicine, Kolkata, India; ^2^Department of Tropical Medicine, School of Tropical Medicine, Department of Health & Family Welfare, Government of West Bengal, Kolkata, India

**Keywords:** PKDL, MAC, POLY, LC-MS/MS, CICs, proteomics, glycated biomarker

## Abstract

**Background:** Post Kala Azar Dermal Leishmaniasis (PKDL) is a non-fatal dermal sequel of Visceral Leishmaniasis (VL), affecting individuals worldwide. Available diagnostic tools lack sensitivity and specificity toward identifying macular (MAC) PKDL patients, due to low parasite load in patients' sample. Confirmatory test like punch biopsy are invasive and painful. Considering the rural nature of this disease and the prevailing situation of diagnostic scenario, PKDL patients mostly remains unattended from receiving proper medical care. They in turn act as “mobile parasite reservoir,” responsible for VL transmission among healthy individuals (HI). This study aims to identify PKDL disease specific glycated protein biomarkers, utilizing the powerful LC-MS/MS technology, which is the tool of choice to efficiently identify and quantify disease specific protein biomarkers. These identified PKDL disease specific novel glycoproteins could be developed in future as immunochromatographic based assay for efficient case detection.

**Methodology:** Previously our lab had identified importance of glycated (Circulating Immune Complexes) CICs, among PKDL patients. This study aims to further characterize disease specific glycated protein biomarkers, among MAC PKDL patients for both diagnostic and prognostic evaluation of the disease. LC-MS/MS based comparative spectral count analysis of MAC PKDL to polymorphic (POLY) PKDL, HI, and Cured (CR) individuals were performed. Proteins level alterations among all study groups were confirmed by Western blot and enzyme-linked immunosorbant Assay (ELISA).

**Results:** Among MAC PKDL patients 43, 60, 90 proteins were altered compared to POLY PKDL, HI, and CR groups, respectively. Filtering for the most significant proteins, Plasminogen (PLG) and Vitronectin (VTN) were identified which promisingly identified MAC PKDL cases. Active surveillance results from endemic districts of West Bengal revealed drastic rise of MAC PKDL cases, alarming the urgency for field adaptive efficient biomarker.

**Conclusion:** This current study aims to establish PLG and VTN as novel diagnostic and prognostic protein biomarker for MAC and POLY PKDL cases management.

## Introduction

PKDL is a non-fatal dermal sequel of VL, caused by protozoan parasite *Leishmania donovani*. On the global scenario, PKDL in mainly restricted to distinct zones of South Asia (India, Nepal and Bangladesh) and East Africa, mainly Sudan. In India, the most prominent fatal form of leishmaniasis present is VL, affecting entirely the eastern region of the country (Zijlstra et al., 2000; WHO, 2010; Desjeux and Ramesh, 2011; Alvar et al., 2012; Perry et al., 2013) The clinical presentation of VL and PKDL differ substantially; where VL patients suffer from sustained fever, hepatosplenomegaly, weight loss and anemia; PKDL patients on the other hand suffer from dermal lesions usually manifested in the form of MAC, Nodular and POLY. The lesion usually appear on face and subsequently gets spread to extremities. The predominance of PKDL patients suffering with MAC lesions varies among different regions; for example in Sudan, PKDL patients suffering with MAC lesions were reported to be 9% whereas in Indian scenario the percentage of PKDL patients suffering with MAC lesions ranges from 15 to 31%. The percentage of PKDL patients suffering with POLY lesions ranges from 69 to 85% in Indian scenario (Ganguly et al., 2010). Although, compared to VL, the mortality rate of PKDL patients is significantly lower, yet PKDL is observed as a stigmatizing disease that brings a substantial socioeconomic burden, further intensified by a hesitancy of patients, to obtain treatment, or due to noncompliance. Lesions, especially the POLY forms, are parasite-rich, concluding that PKDL patients plays a crucial role in the anthroponotic transmission of VL. PKDL patients suffering with MAC lesions, have comparatively lower parasitic load, and thus lack detection sensitivity with standard techniques like rK39 (73% sensitivity) (Salotra et al., 2001) Leishmanin skin test (54% sensitivity), histopathological studies with patients' skin biopsy sample (7–33% sensitivity) (WHO, 2015) and is often misdiagnosed as Vitiligo cases (Domínguez and Toraño, 1999; Jaiswal et al., 2018). In order to completely eliminate Kala-azar in Southeast Asia, proper identification of various routes responsible for the spread of the disease is essential (Zijlstra et al., 2003), which could efficiently distinguish between active PKDL patients and CR individuals as well as between active PKDL patients and other cross disease like Leprosy and Vitiligo. Most of the diagnostic methods for PKDL disease identification are based on highly invasive tissue biopsy methods (WHO, 2015) followed by PCR/qPCR; which requires high technical expertise. Till now very less work has been done toward identification of PKDL disease specific glycated CICs proteome biomarker from patients' plasma. Recent reports by active surveillance have suggested huge predominance of MAC PKDL cases, with very low parasitic load, and acts as efficient reservoir for spread of VL (Sengupta et al., 2018). Thus, there exists an urgent need for biomarkers for early MAC PKDL case detection.

Recent investigations from our group have demonstrated the importance of glycated CICs among PKDL patients (Jaiswal et al., 2018). Integral binding of antibody and antigens leads to the formation of Immune complexes (ICs). Inside human system, these ICs are subjected to various immunological responses like complement deposition, opsonisation (Domínguez and Toraño, 1999), phagocytosis or protease mediated cleaving. ICs accumulation inside patients' body results in various pro-inflammatory effects, including complement activation and cytokine secretion. Deposition of ICs inside tissue and vessel walls leads to inflammation, tissue damage and several other disease manifestations (Hoiby et al., 1986). ICs also induce various pro-inflammatory or anti-inflammatory cytokines which affect disease progression and outcome in several disorders (Rönnelid et al., 2003; Mathsson et al., 2006). ICs acts as complement activators, resulting in their solubilization and prevention of immune precipitation followed by their clearance through erythrocyte complement receptor 1 (CR1). Previous reports have repeatedly suggested association of parasite infections with high levels of CICs which directly leads to tissue damage (Moulds et al., 1992). Previous studies from our lab have reported the presence of high levels of CICs among PKDL patients (Datta et al., 2015; Jaiswal et al., 2018). Further high-throughput studies are required to identify the specific peptide fragments from PKDL patients' CICs, representing specific proteins, which could act as glycated CICs proteome biomarker for both diagnostic and prognostic care of PKDL patients after treatment completion.

Recently, experimental proteomics studies have been applied greatly for studying the pathophysiology of complex diseases, including both infectious and non-infectious ones. Few studies on Leishmaniasis have identified proteomic alteration in the plasma samples of VL patients compared with HI and reported significant alterations of plasma proteins (Rukmangadachar et al., 2011; Bag et al., 2014). Two comparative proteomics studies employing either quantitative (Rukmangadachar et al., 2011) or qualitative (Bag et al., 2014) approach, have reported 26 and 39 differentially expressed protein spots. Various acute-phase proteins were reported to be expressed differentially in VL patients compared to HI. Qualitative study reported up-regulation of α-1-acid glycoprotein and C1 inhibitor; and down regulation of retinol binding protein, whereas quantitative study reported up-regulation of α-1-antitrypsin, α-1-B glycoprotein, and amyloid-A1 precursor; and down regulation of vitamin-D binding protein. These studies highlight the role of proteomics in identifying disease specific protein markers that can help in detection of VL, when parasite load is scanty. Previous reports from our lab have reported altered CICs glycosylation status of PKDL patients (Datta et al., 2015; Jaiswal et al., 2018). High throughput proteomic analysis of PKDL patients' glycated CICs will assist in the identification of major PKDL disease specific protein biomarkers among Indian (MAC and POLY) PKDL population.

## Method

### Study Area and Population

**Figure 1:**
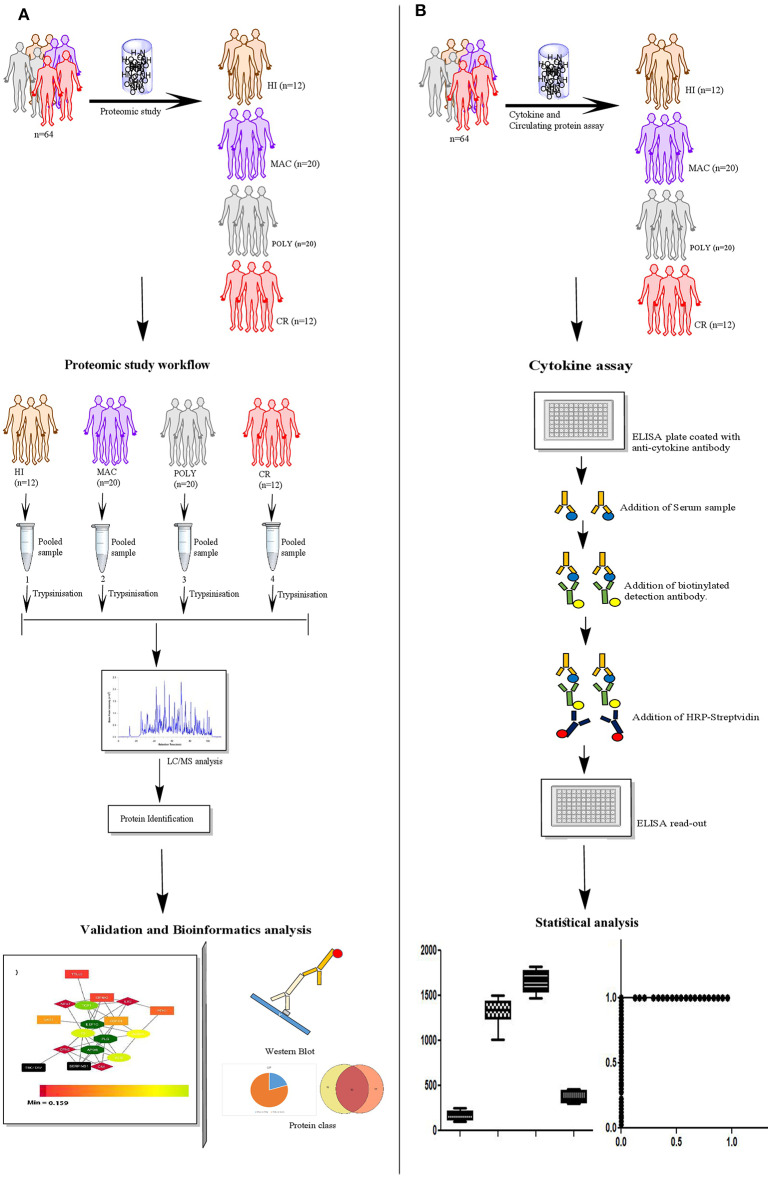
Schematic representation of the experimental study design. **(A)** Sample selection strategy for the proteomics study; HI (*n* = 12), MAC (*n* = 20), POLY (*n* = 20) and CR (*n* = 12). **(B)** Experimental design used for the ELISA; HI (*n* = 12), MAC (*n* = 20), POLY (*n* = 20) and CR (*n* = 12). Patient population used for protein levels, belongs to the same patient population as that of the proteomics study. HI, Healthy Individuals; MAC, Macular PKDL patients; POLY, Polymorphic PKDL patients; CR, Cured Individuals.

PKDL study subjects were enrolled through the ongoing VL Elimination programme (2015-2016) at block and sub-block levels in the state of West Bengal, India. The study subjects recruited were mainly from endemic zones of India namely *Malda and Darjeeling*. Taken together, 20 MAC PKDL patients, 20 POLY PKDL patients and 12 CR individuals were enrolled after taking thorough clinical examinations, information regarding history of VL and rk39 strip test positivity. Exclusion criteria included patients with underlying medical disorders e.g., Heamatological malignancies, Bleeding disorders, chronic liver disease, Diabetes mellitus, severe cardiorespiratory disease, renal disease. Cured (CR) group is described as individuals who have previously received treatment for PKDL and presently they are not suffering from any lesion severity. Additionally, 20 VL patients and 12 HI subjects were also enrolled for the study. Schematic representation of the quantitative proteomics study design by LC-MS/MS, ELISA and Western Blot is provided in the graphical abstract (Figure 1). Disease controls included subjects with similar disease manifestations like Vitiligo (*n* = 12) and Leprosy (*n* = 12), were enrolled from outdoor/indoor department of Dermatology and Tropical Medicine, School of Tropical Medicine, Kolkata.

### Ethics Statement

After primary screening, 2 ml of venous blood was collected in heparinized vials. rK39 strip test was performed. For performing rK39 strip test, the following steps were done. 2ml of venous blood was collected in heparinized vials. rK39 strip test (Kala Azar ^TM^ Rapid Test InBios International, Seattle, WA, USA) as per Govt. guidelines, was performed by trained field medical assistants, according to the standardized protocol (as described by manufacturer). The ethical considerations at the study sites; both at the outdoor and indoor Departments of Dermatology and Tropical Medicine, Calcutta School of Tropical Medicine were reviewed and approved by the Clinical Research Ethics Committee of School of Tropical Medicine, Kolkata (CREC-STM/273 dated: 18.09.2015).

### Sample Preparation

PKDL patients' plasma samples with different lesion gradations i.e., MAC, POLY and CR were pooled down separately; irrespective of gender biasness, along with HI to reduce sample complexity. Briefly, plasma from MAC PKDL (*n* = 20), POLY PKDL (*n* = 20) and CR (*n* = 12) individuals were pooled separately and CICs were precipitated by 50% saturated solution of ammonium sulfate as described by Moss et al. (1982). Next, the samples were dialyzed, using Dialysis tubing (Sigma-Aldrich), with repeated phosphate buffer saline (PBS) changes of 1000 ml for 48 h at 4°C.

### Soluble *Leishmania donovani* Antigen (LDA) Preparation

Crude antigen preparation was performed with *L. donovani* strain MHOM/IN/83/AG83 after culturing them at log growth phase. Promastigotes (1 × 10^7^cells/ml) were harvested in PBS solution (0.02M) and the pellets resuspended in lysis buffer (20 mMTris-HCl, 40 mM NaCl, pH 7.4) containing 2 mM PMSF, 1 mg/ml leupetin, 5 mM EDTA and 5 mM iodoacetamide. The lysates were preserved in −20°C until further use (Blum et al., 2001).

### Parasite ELISA

For determination of anti-leishmanial antibody titer (IgG), parasite ELISA was performed (Bandyopadhyay et al., 2004). Briefly, soluble *L. donovani* antigen in PB (0.02 M, pH 7.2) was used as coating antigen at optimum concentration (1 μg/100 μl/well). After blocking non-specific sites, patient plasma (diluted 1:500; 100 μL/well) were added and incubated for 2 h at 4°C. The wells were then incubated for 1 h at RT with HRP-conjugated anti-human monoclonal IgG (1:15,000) (Sigma-Aldrich, Cat**#**:A0170), to measure the levels of IgG anti-leishmanial antibodies with Tetramethyl benzidine (TMB). Optical density (OD) was measured at 450 nm on micro plate reader (BIORAD PR4100).

### Depletion of High Abundance Proteins

Depletion columns resins were used to remove high abundant plasma proteins to facilitate the detection of low abundant proteins. In this manner, high abundant protein like albumin was removed using Albumin Depletion kit (Pierce−85160) as per the manufacturer's instructions.

### Isolation of Plasma Glycoproteins

Albumin depleted samples were treated with Glycine—HCL buffer as described by Jaiswal et al. (2018), to dissociate the antigen-antibody complex (Jaiswal et al., 2018). Next the acid dissociated CICs were affinity purified using equilibrated Protein A Sepharose 4B column (Invitrogen, USA), at room temperature (RT) for 45 minutes. Antibody free CICs' antigen fraction were collected and subjected to m-phenyl boronic acid column (sigma), overnight at RT, to purify the glycated CICs antigenic fraction (Stoll and Hounsell, 1987; Hageman and Kuehn, 1992). The Glycated fractions were eluted with 50 mM Taurin-NaOH solution, pH 8.7 (Bassil et al., 2004) and were subsequently dialyzed using Dialysis tubing (Sigma-Aldrich) with repeated PBS changes of 1,000 ml for 24 h at 4°C. Protein concentration was measured by Lowry method (Lowry et al., 1951).

### De-glycosylation of Affinity Purified CIC Antigens

To characterize and reconfirm the glycation status, present in the affinity purified CIC antigens, the above column purified samples were de-glycosylated using *Arthrobacter ureafaciens* neuraminidase (Sigma-Aldrich) treatment according to manufacturer's instruction. Briefly, 50 μg of affinity purified CICs antigens were treated with 2 sigma units of neuraminidase in reaction buffer and incubated at 37°C for 3 h. The reaction mixtures were heated at 100°C for 5 min, followed by treatment with 5 units of N-Glycosidase F (Roche, USA), in reaction buffer (20 mM sodium phosphate, 10 mM EDTA, 0.5% (w/v) CHAPS and 0.05% (w/v) SDS), and incubated for 18 h at 37°C. Reactions were ultimately stopped by heating at 100°C for 5 min (Das et al., 2003).

### Lectin Dot Blot

Glycation status of affinity purified CIC antigens were analyzed by DIG Glycan differentiation kit (Roche, USA) according to manufacturer's instruction. Briefly, 5 μg of affinity purified CIC antigens; both enzyme treated and untreated, as described above; were transferred to PVDF membrane followed by Lectin dot blot using DIG Glycan differentiation kit. Membranes were blot dried and analyzed (Sata et al., 1990).

### Buffer Exchange of Affinity Purified CIC Fractions and In-Solution Digestion

Buffer exchange of quantified glycated CIC antigenic fractions, from PBS to triethylammonium bicarbonate buffer (0.5 M) was performed, using Amicon Ultra 0.5 mL Centrifugal filters, as previously described by Ray et al. (2012). In-solution digestion of the CIC fraction was performed by the addition of 1 μL denaturing solution containing 2.0% SDS to 45 μg of CIC samples. The CIC samples were incubated for 1 h at 60°C after subsequent addition of 2 μL of 50 mM reducing reagent (tris (2-carboxyethyl) phosphine). One microliter of cysteine blocking solution (iodoacetamide 100 mM) was added to the CIC samples and incubated for 10 min at RT. Next the CIC samples were treated with Trypsin (Pierce; Thermo Scientific), at a ratio of 1:30 (trypsin: protein) and incubated overnight at 37°C.

### Zip-Tipping and LC-MS/MS

Protein samples were dried by speed-vacuum centrifugation and reconstituted in 40 μL of 0.1% formic acid eluted with acetonitrile and then dried. Samples were reconstituted in 15 μL of 0.1% formic acid. Prior to MS, different peptides present in polypeptide solution were separated with nano LC. Peptides were analyzed by electrospray ionization mass spectrometry using the Easy nano LC 1200 system [Thermo Scientific] coupled to a Q-Exactive Plus Orbitrap mass spectrometer [Thermo Scientific]. Briefly, tryptic digested peptides (6 μl injection volume) were loaded onto a nano-precolumn (Thermo Fisher Scientific Acclaim) PepMap 100 μm × 2 cm, nanoviper C18, 5 μm, 100A. Peptides were separated by a nano HPLC column ES 803 PepMap RSLC C18 2 μm, 100A, 75 μm × 50 cm, and separated with a linear gradient of water/ 80% acetonitrile/0.1% formic acid (v/v) and ion-sprayed into MS with a spray voltage of 1.90KV, capillary temperature 300°C. Further, raw files were analyzed to identify proteins of interest using Proteome Discoverer Software 2.2 (Thermo Scientific); with the following parameters: database; SequestHT Uniprot Homo sapiens database, Type of search; MS/MS Ion Search, Enzyme; Trypsin, Variable modifications; Oxidation (M), Mass values; Monoisotopic, Protein Mass; Unrestricted, Precursor Mass Tolerance; 10 ppm, Fragment Mass Tolerance; 0.05Da, Max Missed Cleavages; 2 and Instrument type; default. Searches were performed with the label-free quantification option selected (Nguyen et al., 2013). The liquid chromatography mass spectrometry (LC-MS/MS) proteomics data were deposited at the Proteome Xchange Consortium through the PRIDE partner repository. Data are available via Proteome Xchange with identifier numbers PXD012960 and 10.6019/PXD012960.

### Determination of Circulating Proteins Titres

Circulating glyco-proteins, PLG and VTN were reported to interact directly with *L. donovani* parasite (Fatoux-Ardore et al., 2014). To evaluate their levels in PKDL infections, stored plasma of PKDL patients were thawed and levels of PLG and VTN were evaluated using standard ELISA kits (Ray Biotech, Suite, Georgia). The tests were carried out as per manufacturer's instruction. Samples used for this assay includes HI, MAC, POLY, CR, active VL, Vitiligo and Leprosy.

### Western Blot Analysis

Affinity purified CICs antigens (30 μg) from plasma of PKDL patients were electrophoresed in SDS PAGE as described by Jaiswal et al. (2018). Samples were ran on 7.5% resolving gel prepared by 30% acrylamide and bisacrylamide solution, 1.5M Tris-HCl (pH-8.8), 10% SDS, 10% APS, and 5 μl TEMED. Electrophoresis was performed on mini protean tetra cell (Bio-Rad, USA) using electrophoresis buffer (Tris 0.025M, glycine 0.19M and SDS 0.1%). Western blot was performed using monoclonal biotin tagged PLG and VTN anti-human antibody (BIOSS, USA) followed by treatment with HRP- avidin anti-human IgG secondary antibody monoclonal IgG (1:1000) (Sigma-Aldrich, Cat**#**:A0170) and developed with DAB substrate kit (Pierce, USA) according to manufacturer's instruction (Singh et al., 2005).

### Statistics

Values for each set of experiment were analyzed by calculating their respective medians and interquartile ranges (IQRs). Data representation was done by Box plots to show Tukey whiskers values, median and IQR. At first, all data sets were subjected for normality test using D'Agostino & Pearson omnibus normality test. Univariate non-parametric ANOVA analysis (i.e., Kruskal Wallis test) was performed and if data represents significant value, *post-hoc* (dunn) test was implemented for defining differences within each group with *p*-values smaller than 0.01 were statistically significant. Cut-off value of circulating glycoproteins (PLG and VTN) was determined by using Youden index. Efficiency of levels of circulating glycoproteins (PLG and VTN) and anti-leishmania antibody titer (IgG), for prediction of MAC PKDL and its further progression, were analyzed using receiver operating characteristic (ROC) curves. Sensitivity and specificity values for the same were calculated at different threshold points. Areas under curve (AUC) values were also calculated, which were statistically significant. Statistical analysis was performed using the Graph-Pad Prism statistics software (Graph-Pad Software Inc., San Diego, CA) and SPSS Inc (Chicago, IL).

### Protein Network Analysis

Human gene symbols for identified proteins after LC-MS/MS analysis were uploaded to web-based STRING tool (version 10.5) to identify functional protein association network (Shannon et al., 2003). Gene ontology analysis was performed to retrieve overrepresented biological process terms (Maere et al., 2005).

## Result

### Plasma Proteomic Changes

**Figure 2:**
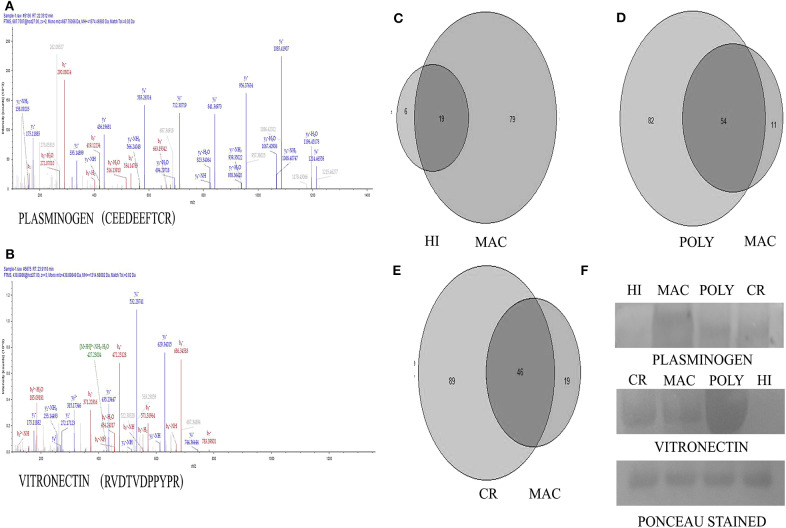
Representative MS-MS spectra of significantly altered proteins obtained from Q-ExactivePlus Orbitrap mass spectrometer study. **(A)** MS-MS spectra of Plasminogen (PLG) protein. **(B)** MS-MS spectra of Vitronectin (VTN) protein. **(C)** Venn diagram showing number of common proteins in all the patients with MAC lesions compared to HI. **(D)** Venn diagram showing number of common proteins in all the patients with MAC lesions compared to POLY lesions. **(E)** Venn diagram showing number of common proteins in all the patients with MAC lesions compared to CR individuals. **(F)** Validation of Plasminogen and Vitronectin using western blotting image Ponceau stained image of the blot after transfer.

Plasma samples from PKDL patients with MAC (*n* = 20), POLY (*n* = 20), CR (*n* = 12) and HI (*n* = 12) were isolated and processed for proteomics analysis, involving in-solution digestion, followed by analysis on a nano LC-MS/MS platform including an Q-Exactive Plus orbitrap mass spectrometer. Samples were arranged in groups: (i) MAC vs. HI, (ii) MAC vs. POLY and (iii) MAC vs. CR to identify relative abundance of proteins. All samples were run in triplicates. PKDL patients and HI characteristics are presented in Table 1. Of these 11, 42 and 19 proteins with > 2 peptide matches were differentially down-regulated in MAC vs. HI, MAC vs. POLY and MAC vs. CR, respectively and 31, 18 and 70 proteins were differentially up-regulated in MAC vs. HI, MAC vs. POLY and MAC vs. CR, respectively. Individual fold change values for identified proteins among all the patient groups. Proteins common in all the study groups; MAC vs. HI, MAC vs. POLY and MAC vs. CR; were chosen for further analysis to identify disease specific biomarkers that will allow the diagnosis as well as PKDL progression. Interestingly, several proteins, such as Profilin-1 and Alpha-2-macroglobulin were up-regulated (fold change >1.2) in all the study groups. Protein such as Galectin-1, Protein S100-A14, 60S Ribosomal protein L11, 40S ribosomal protein S2 and Fascin were up-regulated (fold change >1.2) in MAC vs. HI and MAC vs. CR whereas 60S Ribosomal protein L-37A was down-regulated (fold change <0.98) in MAC vs. POLY. Also proteins like Prothrombin, Fructose-bisphosphatealdolase, NF45 and Elongation factor-1 gamma were up-regulated (fold change >1.2) in MAC vs. POLY and MAC vs. CR groups. Proteins such as keratin type 1 cytoskeleton 14, Alpha-enolase and Galectin 7 were down-regulated (fold change <0.98) in MAC vs. HI and MAC vs. POLY group, whereas proteins such C4b-binding protein alpha chain, Keratin, type II cytoskeletal 2 epidermal, Fibronectin, Keratin, type I cytoskeletal 9, Clusterin, Afamin, Complement C5, Fibrinogen gamma chain, Hemopexin and Apolipoprotein A-II were down-regulated (fold change <0.98) in MAC vs. POLY and MAC vs. CR groups. Proteins such as PLG were significantly up-regulated among PKDL patients with MAC lesions as compared to PKDL patients with POLY lesions, HI and CR individuals whereas VTN is significantly up-regulated among PKDL patients with POLY lesions as compared to PKDL patients with MAC lesions, HI and CR individuals (Figure 2). Identification of *L. donovani* specific proteins were not included in the study as the main aim of the study was to identify proteomic alterations in the glycated CICs isolated from the plasma of PKDL patient in comparison to HI and CR individuals.

**Table 1:** Patient and Healthy control characteristics.

	**Patients (*n* = 52)**	**Control (*n* = 12)**
Age		
Years (SD)	26.08 (16.26)	27.78 (7.78)
Gender		
Male	25	7
Female	27	5
Type of PKDL patient		
Macular	20	_
Polymorphic	20	_
Cured	12	_
Previous VL history	52	_

### Comparison of Anti-Leishmanial Antibody Titres Among PKDL Patients and Control Individuals

**Figure 3:**
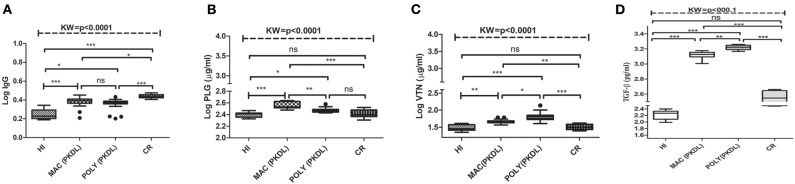
Box plot of plasma level of **(A)** IgG **(B)** PLG (μg/ml) **(C)** VTN (μg/ml) **(D)** TGF-β (pg/ml) for HI, MAC PKDL patients, POLY PKDL patients and CR individuals. Whiskers calculated adopting the Tukey method. Outliers showed as dots. *p < 0.01, **p < 0.001, ***p < 0.0001, ns-not significant: Kruskal–Wallis test (K) followed by Dunn's multiple comparison test.

*Leishmania* infection mounts humoral responses against *Leishmania* antigen in the form of immunoglobulins of isotypes IgG, which are important serological markers in the host. To assess the status of primary immunoglobulin responses, present study investigated IgG, antibody titres in the plasma of PKDL patients with respect to HI and CR individuals. Parameters involving plasma dilutions and antigen concentrations were standardized to set optimal ELISA condition for the serological assay. Anti-leishmanial antibody titres of IgG in plasma of PKDL patients showed enhanced responses. The performances of the sensitivities and the specificities of the ELISA with plasma from patients with PKDL were also evaluated. The reactivity of the IgG, antibodies from the plasma of patients with PKDL (*n* = 40) were significantly higher than those of the plasma from the HI but lower than CR (*p* < 0.0001). The “mean ± SEM” of anti-leishmanial IgG antibody titer in the plasma of MAC and POLY PKDL patients were 2.414 ± 0.062 and 2.275 ± 0.065 respectively in comparison to HI (1.772 ± 0.062) and CR individuals (2.733 ± 0.039) (Figure 3A). The sensitivity, specificity and AUC values for IgG ELISA among various experimental groups i.e., MAC vs. HI, MAC vs. POLY and MAC vs. CR is represented in **Table 3**. The respective AUC values for different experimental groups showed poor performance of IgG ELISA, in comparison to glyco-proteins PLG and VTN, for discriminating PKDL cases from controls (**Figures 5A–I**).

### Protein Levels in Individual Samples

**Figure 4:**
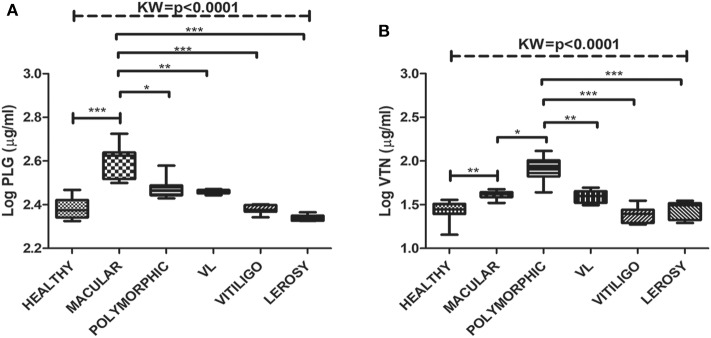
Box plot of plasma level of **(A)** PLG (μg/ml) **(B)** VTN (μg/ml) for HI, MAC PKDL patients, POLY PKDL patients, Vitiligo and Leprosy patients. Whiskers calculated adopting the Tukey method. Outliers showed as dots. *p < 0.01, **p < 0.001, ***p < 0.0001, ns-not significant: Kruskal-Wallis test (K) followed by Dunn's multiple comparison test.

Significant variations in the levels of circulating glyco-proteins like PLG and VTN, were analyzed in individual samples of MAC (*n* = 20), POLY (*n* = 20), CR (*n* = 12), HI (*n* = 12), active VL (*n* = 20), Leprosy (*n* = 12) and Vitiligo (*n* = 12) by ELISA. PLG levels were significantly up-regulated in MAC with “mean ± SEM” value of 368.3 ± 11.48, as compared POLY (297.3 ± 6.387), CR (261.1 ± 12), HI (250.9 ± 7.080) (Figure 3B), active VL (286 ± 1.32), Vitiligo (239.6 ± 2.75) and Leprosy (218.17 ± 1.82) (Figure 4A) whereas VTN levels were up-regulated in POLY “mean ± SEM” value of 65.84 ± 4.835 as compared to MAC (46.64 ± 1.378), CR (32.44±1.751) and HI (30.46±1.88) (Figure 3C), active VL (38.95 ± 1.15), Vitiligo (24.75 ± 1.56) and Leprosy (28.25 ± 1.72) (Figure 4B).

**Figure 5:**
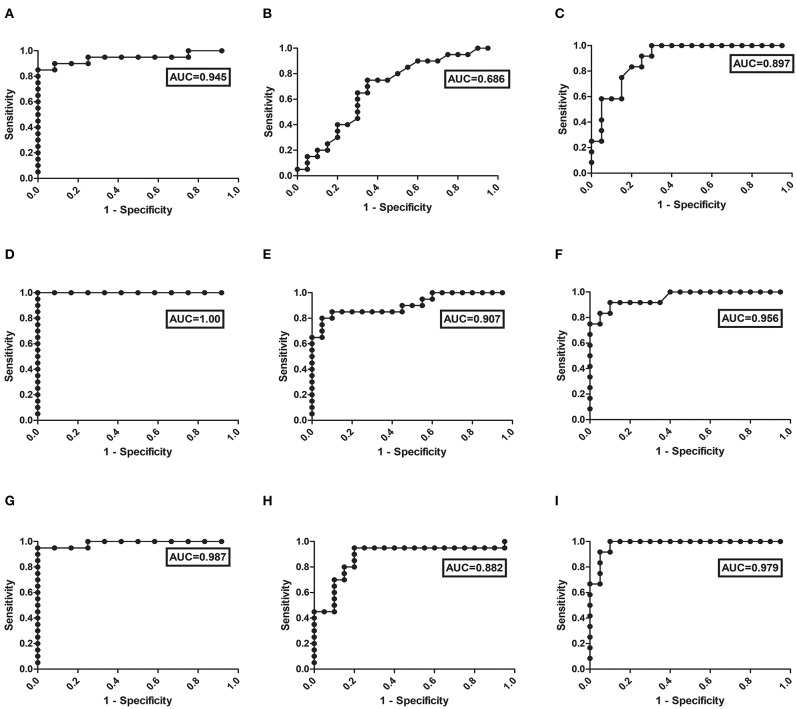
ROC curve obtained from the ELISA values for the detection of: (I) IgG in different experimental groups i.e., **(A)** MAC vs. HI, **(B)** MAC vs. POLY, **(C)** MAC vs. CR; (II) PLG in different experimental groups i.e., **(D)** MAC vs. HI, **(E)** MAC vs. POLY, **(F)** MAC vs. CR; and (III) VTN in different experimental groups i.e., **(G)** MAC vs. HI, **(H)** MAC vs. POLY, **(I)** MAC vs. CR; from the plasma of PKDL patients' samples.

### Functional Data Analysis of Differentially Expressed Proteins

**Figure 6:**
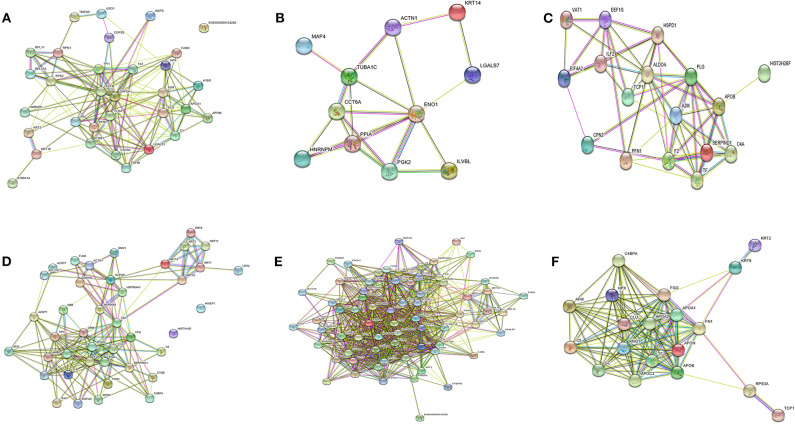
Protein-protein interaction networks. Protein-protein interactions of differentially expressed protein in different study groups. **(A)** Up regulated proteins in MAC PKDL patients compared to HI individuals. **(B)** Down regulated proteins in MAC PKDL patients compared to HI individuals. **(C)** Up regulated proteins in MAC PKDL patients compared to POLY PKDL patients. **(D)** Down regulated proteins in MAC PKDL patients compared to POLY PKDL patients. **(E)** Up regulated proteins in MAC PKDL patients compared to CR individuals. **(F)** Down regulated proteins in MAC PKDL patients compared to CR individuals. Purple and green colored lines for experimental and textmining evidence of different interactions.

To identify the biological functions, associated with the proteins that exhibited changed abundance in plasma of PKDL patients with MAC and POLY lesion manifestations as compared to HI and CR individuals, we performed STRING and Cytoscape analysis. Protein network were generated for the protein sets of all the groups (MAC vs. HI, MAC vs. POLY and MAC vs. CR) using STRING database (Figure 6). Gene ontology mining of the identified proteins revealed alteration in the biological functions, involved mostly in inflammatory and immune response and transporter activity (Table 2). List of various up-regulated and down-regulated proteins in various study groups i.e., MAC vs HI, MAC vs POLY and MAC vs CR are provided in Tables S1–S6.

**Table 2:** Overrepresented gene ontology (biological process) terms associated with differential proteins in MAC PKDL patients compared to POLY PKDL patients.

**Protein**	**# Proteins regulation in PKDL**	**Biological process**	**Associated molecules**
Down regulated	42	Inflammation response	APOD, C4BPA, C5, KRT1, SERPING1, VTN, FGA, HP, ORM1, FGA, HP, ORM1
		Immune system process	ACTG1, AMBP, APOA2, C1QB, C4BPA, C5, CLU, HPX, HSP90AA1, HSP90B1, KRT1, SERPING1, VTN, APOD, ORM1, C1QB, C4BPA, C5, CLU, SERPING1, GAPDH, AZGP1, FGA, HP, ITGB3
		Transport	ACTG1, AMBP, CLU, FGA, FGG, HBB, HP, HPX, HSP90AA1, HSP90B1, ITGB3, MYH9, SERPING1, VTN, AFM, APOA2, APOD, AZGP1, ORM1, RPL37, TTR
Up regulated	18	Transport	A2M, ALDOA, APOB, C4A, PFN1, PLG, TBC1D17

### Western Blot Protein Validation and Diagnostic Performance of Differentially Expressed Proteins

To confirm the results of proteomic analysis, western blots were performed which validated the presence of differentially expressed proteins, namely PLG and VTN. Western blot analysis was performed on pooled samples of PKDL patients with lesion gradations of MAC (*n* = 20) and POLY (*n* = 20) and among HI (*n* = 12) and CR (*n* = 12) individuals (Figure 2). Ponceau staining of the transferred blots indicated equal loading of each samples in each lane. Consistent with our proteomics findings, western blot analysis revealed PLG to be over-expressed in MAC patients compared to POLY patients, HI and CR individuals, whereas VTN was found to be significantly over-expressed in POLY patients as compared to MAC patients, HI and CR individuals.

To evaluate the individual performance of the circulating glyco-proteins PLG, VTN as efficient PKDL specific biomarkers, ROC curve was performed (Figures 5D–I). The area under the ROC curve (AUC) is a measure of how accurately the circulating glyco-proteins can distinguish between MAC and HI; MAC and POLY; MAC and CR. AUC values of PLG and VTN is represented in Table 3, which shows better performance of glycoprotein PLG and VTN as PKDL specific biomarkers in comparison to IgG based ELISA.

**Table 3:** Sensitivity, Specificity and AUC value of IgG, PLG and VTN in different experimental groups of PKDL patients: MAC vs HI, MAC vs POLY and MAC vs CR.

	**IgG**			**PLG**			**VTN**		
	**MAC vs HI**	**MAC vs POLY**	**MAC vs CR**	**MAC vs HI**	**MAC vs POLY**	**MAC vs CR**	**MAC vs HI**	**MAC vs POLY**	**MAC vs CR**
Sensitivity (%)	85	75	100	95	85	91.67	95	95	100
Specificity (%)	100	65	70	100	90	90	100	80	90
AUC	0.945	0.686	0.897	1.00	0.907	0.956	0.987	0.882	0.979

### Prognostic Potential of Glyco-Protein PLG and VTN vs. Conventional Parasite ELISA

To evaluate the prognostic potential of glyco-protein PLG, VTN and anti-leishmanial antibody ELISA, longitudinal monitoring was performed among 10 paired PKDL samples at day 0 and >180 after drug administration. The glyco-protein PLG and VTN showed that all the 5 patients after treatment with Miltefosine (drug responsive group) demonstrated 2 fold decrease in the values (for PLG; *p* = 0.0028 and for VTN; *p* = 0.0041), indicating that these patients were responding to treatment, which was also confirmed clinically. Further, PLG and VTN glycoproteins could also identify 5 drug unresponsive patients (receiving LAmB) (for PLG; *p* = 0.0191 and for VTN; *p* = 0.0252) thus, indicating its utility for therapeutic monitoring. In comparison, the conventional anti-leishmanial antibody based ELISA failed to differentiate follow-up from baseline cases in both drug responsive and unresponsive groups, where the mean titer showed insignificant differences for both drug responsive (*p* = 0.378) and drug unresponsive (*p* = 0.129) cases.

## Discussion

PKDL patients are considered as an important reservoir of *L. donovani* parasite, responsible for VL infection (Molina et al., 2017). PKDL patients residing in the endemic zones of West Bengal, are mostly from poor background and they show negligence toward default treatment due to two major concern; (i) the diagnostic test are highly invasive, painful and results in cosmetic scars, (ii) the mortality rate from PKDL disease is very less. Thus, absence of non-invasive and highly efficient diagnostic method for PKDL case detection, leaves most of these patients unattended. These PKDL patients acts as parasite reservoir for spread of VL. This lack of efficient diagnostic methods and the negligence shown by PKDL patients toward treatment creates a huge socio-burden, which requires immediate action (Basher et al., 2012) in order to totally eliminate Kala Azar. For efficient identification and treatment of PKDL patients, confined to the endemic zones, government initiative has started since 2005, The Kala Azar Elimination Program, as a joint venture between India, Bangladesh and Nepal (Zijlstra et al., 2017). Recent report by Molina et al. (2017) and Moulik et al. (2018) suggested higher and/or equal infective potential and prevalence of PKDL patients suffering with MAC lesions, in contrast to PKDL patients suffering with POLY lesions, in endemic zone of West Bengal. The gold standard diagnostic approach, slit skin smear test shows very low parasite detection sensitivity, which ranges from 4 to 58% (Mondal et al., 2010; Zijlstra, 2016) for PKDL patients suffering with MAC lesions. Further most MAC PKDL patients are often misdiagnosed as pityriasis versicolor, due to similar dermal manifestation and very low number of LD bodies which is often missed out from detection. This prevents MAC PKDL patients from receiving prescribed diagnostic care (Das et al., 2011; Singh et al., 2015). Recent report suggested that PKDL patients suffering with MAC lesions harbor sufficient parasites, which in turn are transmitted by sandfly to HI (Moulik et al., 2018) resulting in active VL cases. The present available diagnostic methods fail to differentiate between fresh PKDL patients with active MAC lesions in comparison to CR individuals, after treatment completion.

The later situation arises due to persistence of high titres of anti-leishmanial antibody even after treatment completion (Desjeux et al., 2013). Moreover anti-leishmanial drugs requires longer duration, to reach the dermal site, due to partial skin diffusion (Zijlstra et al., 2017). The prescribed drugs of choice; Miltefosine and liposomal amphotericin B (LAmB), both lack efficacy toward parasite clearance. Miltefosine has been reported previously to show high efficacy toward parasite clearance in PKDL affected patients, but recent studies reported decline in its effectiveness in parasite clearance (Ghosh et al., 2015; Ramesh et al., 2015). Although LAmB has shown high efficacy in VL treatment, the drug efficacy in parasite clearance, especially for PKDL patients suffering with MAC lesions, is remarkably very low (Moulik et al., 2018). For proper disease administration, both diagnostic and prognostic management of PKDL patients stands as a crucial point. The goal of our present study is to identify glycated CICs protein biomarkers, to efficiently combat this gross disease situation.

In the current study, we have shown that the glycated CICs proteome of PKDL patients suffering with MAC and/or POLY lesion, differ significantly compared to HI and CR individuals. In addition, significant difference were observed among the CICs proteome of MAC PKDL patients, compared to POLY PKDL patients. Exploiting the CICs proteome difference associated with different PKDL patients, HI and CR individuals, we were able to identify two differentially expressed glycated CICs protein, that have the potential to act as diagnostic and prognostic protein biomarker for efficient management of PKDL patients.

Previous studies reported the presence of altered proteomic profile among the plasma of VL patients. Plasma serves as a precious source for analysis of pathophysiological alterations associated with both disease identification and progression. Qualitative proteomic study on the plasma samples of VL patients' reported up-regulation of α-1-acid glycoprotein and C1 inhibitor and down regulation of retinol binding protein and transport protein transthyretin (Rukmangadachar et al., 2011), whereas quantitative study reported up-regulation of α-1-antitrypsin, α-1-B glycoprotein, and amyloid-A1 precursor; and down regulation of vitamin-D binding protein (Bag et al., 2014). These proteins were found to play important role in disease pathology, for example α-1-acidglycoprotein is elevated during systemic tissue inflammation and is involved in neutrophil inactivation, chemotaxis, and oxidative metabolism (Pucadyil et al., 2004). The importance of plasma proteome profile for identification of disease specific protein biomarkers among various diseases were previously reported for Dengue patients (Jadhav et al., 2017), for Rheumatoid arthritis patients (Ohyama et al., 2012) and for VL patients (Bag et al., 2014). No such studies were reported previously for PKDL patients. Study of this kind will be of immense importance for the identification of PKDL specific protein biomarker, further providing assistance toward disease progression and monitoring therapeutic response among PKDL patient.

In the present proteomic study targeting the glycated CICs, of PKDL patients, we have identified 32 up-regulated and 11 down-regulated proteins among MAC PKDL patients compared to HI. Similarly, 18 up-regulated and 42 down-regulated proteins were observed among MAC PKDL patients compared to POLY PKDL patients. Moreover, 71 up-regulated and 19 down-regulated proteins were observed among MAC PKDL patients compared to CR individuals. These proteins were largely linked to inflammatory response, immune system process and transport, which could be in accordance with various dermal manifestations linked with PKDL patients suffering with MAC and POLY lesions.

Previous reports have suggested that *Leishmania* parasite interacts with specific host cellular targets (dermal dendritic cells, mast cells and macrophages) (Kaye and Scott, 2011) as well as the promastigotes gets deposited into the dermal extracellular matrix (Moreno et al., 2010), for efficient infection. Recent works by direct binding assay with intact live *Leishmania* promastigotes, revealed the host interacting partners for efficient *Leishmania* infection. These studies identified various molecules, among which circulating glycoproteins PLG and VTN, have shown very high binding affinity with *L. donovani* promastigotes (Fatoux-Ardore et al., 2014). Our experimental data for the first time shows higher expression of circulating glycoproteins PLG and VTN, present in the glycated CICs of PKDL patients' plasma samples, in comparison to that of HI and CR individuals. Glycoprotein PLG is a member of the extracellular matrix (ECM) component and is critical for blood coagulation which assists in neutrophils and macrophage migration during wound healing (Serada et al., 2010).

Recent studies reported the binding of *L. donovani* parasite to PLG and to its activated form, plasmin (Fatoux-Ardore et al., 2014). This interaction is assisted by enolase secreting exocytic vesicle, which in turn traps the macrophages and possibly allows the parasite to migrate to dermis (Levin and Burgner, 2014; Fujimoto et al., 2015). Interaction of PLG with *L. donovani, Leishmania braziliensis, Leishmania tropica, Leishmania major*, and *Leishmania infantum* has recently been reported (Fatoux-Ardore et al., 2014). Studies suggest that the activation of TGF-β depends on availability of various molecules such as plasmin, urokinase plasminogen activator, M6P, etc.; which in turn depends on activation state macrophage (Song and Wang, 2015).

Glycoprotein VTN, found in blood and extracellular matrix, is a multifunctional glycoprotein which binds to glycosaminoglycan, collagen, PLG and urokinase-receptor (uPAR). It is also located in extracellular matrix and binds to PLG activation inhibitor-1, complement, heparin and thrombin-antithrombin III complexes. This results in cascade of immunological response and regulation of clot formation (Kadowaki et al., 2011). uPAR, a VTN binding protein, is found to associate with latent TGF-β and leads to TGF-β conversion, suggesting the role of VTN toward TGF-β availability, activation and degradation (Godár et al., 1999). Recently reported, VTN has been found to interact with parasite *L. donovani* and *Leishmania braziliensis*, indicating its possible role toward host parasite interaction (Fatoux-Ardore et al., 2014).

TGF-β is a growth regulatory protein known for its diversified biological activity like wound healing, growth, and differentiation and angiogenesis. Inside the cell, TGF-β is present in two milieu; latent form and active form (Sporn et al., 1986; Barnard et al., 1990; Massague et al., 1992; Roberts and Sporn, 1993). Previous studies targeting the immunological characterization of active TGF-β revealed its potential role as regulatory cytokine which suppresses iNOS synthase, IFN- gamma production and Th1 and Th2 cell production (Gantt et al., 2003) which in turn assists in survival of *Leishmania* parasite inside the macrophages. Further studies suggested that higher levels of TGF-β causes down regulation of IFN- gammaR among Indian PKDL patients (Ansari et al., 2006, 2008). In our studies, higher levels of TGF-β were observed among PKDL patients suffering from MAC and POLY lesions as compared to HI and CR individuals (Figure 3D).

For the first time, based on the proteomics study supported by western blot and ELISA, we report higher levels of circulating glycoproteins; PLG, VTN and plasma cytokine TGF-β, among PKDL patients with MAC and POLY lesions, compared to HI and CR. TGF-β activation plays an important role for *L. donovani* survival inside macrophages by enhancing the production of IL-10 cytokine (Mukhopadhyay et al., 2014). Previous studies have reported various methods of activation of TGF-β involving either one or both the above two circulating glycoproteins; PLG and VTN. Although no such studies has been conducted to reveal the direct role of these proteins for disease progression among Indian PKDL patients. One study has revealed the binding of PLG and VTN directly on the surface *L. donovani* (Fatoux-Ardore et al., 2014) where as another study has revealed that the activation of TGF-β assist in survival of *Leishmania chagasi* inside macrophages (Gantt et al., 2003).

Taken together circulating glycoproteins PLG, VTN and cytokine TGF-β add up to PKDL disease severity. Based on the importance of PLG, VTN and TGF-β, each contributing individually toward PKDL progression, we studied the association of PLG and VTN with cytokine TGF-β. Positive association was observed between PLG-TGF-β (*p* < 0.0001) and VTN-TGF-β (*p* < 0.0001) which could be used as a prognostic marker for PKDL. TGF-β interacts directly with Plasmin- the activated form of PLG. Thus, our investigation revealed for the first time that circulating glycoprotein PLG and VTN could serve as severity biomarker for PKDL patients along with TGF-β; an important cytokine for PKDL severity, as reported previously by Saha et al. (2007).

Glycoprotein PLG and VTN could successfully differentiate between drug responsive and drug un-responsive PKDL patients. In this study 10 PKDL patients were longitudinally monitored, among which 5 PKDL patients were recognized as clinically CR as no dermal lesions was observed after treatment with Miltefosine. Among these patients the levels of PLG and VTN were significantly reduced and were at par with the PLG and VTN levels of CR individuals. Whereas, 5 PKDL patients treated with LAmB did not show regression of dermal lesion and PLG and VTN levels were at par with the MAC and POLY PKDL patients. The titer value “mean ± S.E.M” value of 405.7 ± 19.96 μg/ml and 236.4 ± 13.42 μg/ml was obtained, for PLG, for drug responsive cases at presentation with respect to follow up. Similarly for VTN, the titer value “mean ± S.E.M” value of 49.33 ± 3.55 μg/ml and 26.65 ± 0.654 was obtained, for drug responsive cases at presentation with respect to follow up. However, for drug un-responsive cases “mean ± S.E.M” value of 425.4 ± 39.04 μg/ml and 306.1 ± 5.246 μg/ml was obtained for PLG at presentation with respect to follow up. Similarly for VTN, the titer value “mean ± S.E.M” value of 70.98 ± 2.171 μg/ml and 60.05 ± 1.644 μg/ml was obtained for drug un-responsive cases, reflecting no significant decrease in titers even after treatment completion. Hence VTN and PLG were able to efficiently identify the drug unresponsiveness of these PKDL patient.

Conventional anti-leishmanial antibody based ELISA failed to efficiently differentiate between drug responsive and unresponsive groups. Due to limited number of PKDL patient samples, we were unable to analyse the effectiveness of these biomarker proteins; PLG and VTN, toward monitoring the specific drug dosage required for individual patient care. Future studies with higher patient pool will reveal the answer.

PKDL patients suffering with MAC lesions are tough to diagnose and are often misdiagnosed as Vitiligo or Pityriasis versicolor, having similar dermal lesions. Due to very less parasite load among MAC PKDL patients, most of the diagnostic tools lack sensitivity toward efficient case detection. The most commonly used rK39 strip test, Leishmania skin test (LST) and histopathological studies with patients' skin biopsy has only 73, 54, and 7–33% sensitivity, respectively toward detecting MAC PKDL patients (Zijlstra et al., 1994; Ramesh and Mukherjee, 1995; Sundar et al., 1998; Sharma et al., 2000; Salotra et al., 2001, 2003) With such difficult scenario to efficiently target and detect these PKDL patients, which in turn acts as a reservoir for transmission of *L. donovani* parasite and target healthy individuals resulting in VL, it becomes very essential to identify these patients efficiently.

To answer the above problem i.e., for efficient detection of PKDL patients suffering with MAC lesions, where parasite load is very less, we compared the differential fold change levels of PLG and VTN among PKDL patients suffering with MAC and POLY lesion. Our experimental LC-MS/MS data revealed up-regulation of PLG and down regulation of VTN among PKDL patients suffering with MAC lesions when compared to PKDL patients suffering with POLY lesions. This reveals that the ratio of VTN vs. PLG could serve as an efficient biomarker for efficient diagnosis for PKDL patients suffering with MAC lesions. Pathway analysis of various differentially expressed proteins, observed during the proteomic study of the glycated CICs of PKDL patients revealed alterations in transportation and immunological pathway (Table 2).

## Conclusion

In conclusion, these novel observations from the proteomic profiling of the glycated CICs of PKDL patients revealed two important circulating glycoproteins PLG and VTN, with significantly higher titers in PKDL diseased individuals (MAC and POLY) in comparison to control groups (HI, VL, CR, Vitiligo, and Leprosy). Hence the above identified proteins have immense potential to act as PKDL diagnostic and prognostic biomarkers. This study opens a broad spectrum for efficient targeted drug designing for successful control and elimination of PKDL.

## Data Availability Statement

The datasets generated for this study can be found in the data are available via ProteomeXchange with identifier PXD012960.

## Ethics Statement

The studies involving human participants were reviewed and approved by Clinical Research Ethics Committee School of Tropical Medicine (CREC-STM) Under Central drugs standard control organisation (CDSCO), Under Directorate General of Health Services, Ministry of Health And Family Welfare, Government of India. The patients/participants provided their written informed consent to participate in this study.

## Author Contributions

PJ and SM contributed to conceptualization, data curation, writing the original draft, and review and editing the manuscript. PJ, GP, MG, BS, and SM were responsible for the formal analysis. SM acquired the funding. PJ, MG, BS, and SM carried out the investigation. PJ worked on the methodology and visualization. MG, BS, and SM were responsible for administration, resources, and supervision. PJ and GP helped with the software and validation.

## Conflict of Interest

The authors declare that the research was conducted in the absence of any commercial or financial relationships that could be construed as a potential conflict of interest.

## Abbreviations

VL: Visceral Leishmaniasis
PKDL: Post Kala Azar Dermal Leishmaniasis
MAC PKDL: Macular PKDL
POLY PKDL: Polymorphic PKDL
CR: Cured individual
HI: Healthy individual
LC-MS/MS: Liquid Chromatography-Mass Spectrometry
CICs: Plasma, Circulating Immune Complexes.
