# Supplementary material for: Clinical Proteomics Profiling for Biomarker Identification Among Patients Suffering With Indian Post Kala Azar Dermal Leishmaniasis
Source: Front Cell Infect Microbiol. 2020 May 27;10:251. doi: 10.3389/fcimb.2020.00251 (PMC7266879; doi:10.3389/fcimb.2020.00251)
Supplement: Supplementary file 1 [file Table_1.DOCX]

**Table S1.** List of up regulated proteins in MAC vs HI individuals

| **Accession number** | **Gene symbol** | **Approved name** | **Fold change (Mac/HI)** | **Coverage** | **No. of peptides** |
| --- | --- | --- | --- | --- | --- |
| P08779 | KRT16 | Keratin, type I cytoskeletal 16 | 1.584 | 9 | 3 |
| P04843 | RPN1 | Dolichyl-diphosphooligosaccharide--protein glycosyltransferase subunit 1 | 100 | 3 | 1 |
| Q9BVK6 | TMED9 | Transmembrane emp24 domain-containing protein 9 | 100 | 9 | 1 |
| P05556 | ITGB1 | Integrin beta-1 | 1.732 | 2 | 1 |
| P60709 | ACTB | Actin, cytoplasmic 1 | 100 | 11 | 2 |
| P49327 | FASN | Fatty acid synthase | 100 | 2 | 2 |
| P35908 | KRT2 | Keratin, type II cytoskeletal 2 epidermal | 100 | 7 | 3 |
| P04406 | GAPDH | Glyceraldehyde-3-phosphate dehydrogenase | 100 | 18 | 2 |
| Q16658 | FSCN1 | Fascin | 100 | 4 | 2 |
| P21333 | FLNA | Filamin-A | 100 | 1 | 2 |
| P08670 | VIM | Vimentin | 100 | 7 | 1 |
| P15880 | RPS2 | 40S ribosomal protein S2 | 100 | 6 | 1 |
| P52597 | HNRNPF | Heterogeneous nuclear ribonucleoprotein F | 100 | 7 | 1 |
| P04217 | A1BG | Alpha-1B-glycoprotein | 100 | 7 | 2 |
| P07737 | PFN1 | Profilin-1 | 100 | 14 | 1 |
| Q15582 | TGFBI | Transforming growth factor-beta-induced protein ig-h3 | 100 | 3 | 1 |
| I3L0A0 | TMEM189-UBE2V1 | HCG2044781 | 100 | 8 | 1 |
| Q9UNS2 | COPS3 | COP9 signalosome complex subunit 3 | 100 | 4 | 1 |
| O00116 | AGPS | Alkyl dihydroxyacetonephosphate synthase, peroxisomal | 100 | 4 | 1 |
| P60174 | TPI1 | Triosephosphate isomerase | 100 | 6 | 1 |
| P29401 | TKT | Transketolase | 100 | 6 | 1 |
| P62913 | RPL11 | 60S ribosomal protein L11 | 100 | 13 | 1 |
| P01024 | C3 | Complement C3 | 100 | 1 | 1 |
| P61513 | RPL37A | 60S ribosomal protein L37a | 100 | 20 | 1 |
| O60763 | USO1 | General vesicular transport factor p115 | 100 | 2 | 1 |
| P01023 | A2M | Alpha-2-macroglobulin | 4.978 | 1 | 1 |
| P02790 | HPX | Hemopexin | 100 | 2 | 1 |
| O95445 | APOM | Apolipoprotein M | 100 | 5 | 1 |
| Q9HCY8 | S100A14 | Protein S100-A14 | 100 | 11 | 1 |
| P02647 | APOA1 | Apolipoprotein A-I | 100 | 4 | 1 |
| P09382 | LGALS1 | Galectin-1 | 100 | 13 | 1 |
| B2R7F8 | PLG | Plasminogen | 100 | 2 | 1 |
