# Supplementary material for: Clinical Proteomics Profiling for Biomarker Identification Among Patients Suffering With Indian Post Kala Azar Dermal Leishmaniasis
Source: Front Cell Infect Microbiol. 2020 May 27;10:251. doi: 10.3389/fcimb.2020.00251 (PMC7266879; doi:10.3389/fcimb.2020.00251)
Supplement: Supplementary file 2 [file Table_2.DOCX]

**Table S2.** List of down regulated proteins in MAC vs HI individuals

| **Accession number** | **Gene symbol** | **Approved name** | **Fold change (Mac/HI)** | **Coverage** | **No. of peptides** |
| --- | --- | --- | --- | --- | --- |
| P02533 | KRT14 | Keratin, type I cytoskeletal 14 | 0.255 | 19 | 6 |
| A0A2R8Y6G6 | ENO1 | Alpha-enolase | 0.547 | 12 | 4 |
| P47929 | LGALS7 | Galectin-7 | 0.094 | 26 | 2 |
| A0A024R694 | ACTN1 | Actinin, alpha 1, isoform CRA_a | 0.068 | 4 | 2 |
| B7Z1K5 | TUBA1C | Tubulin alpha chain | 0.147 | 7 | 2 |
| A0A140VJR3 | PGK2 | Phosphoglycerate kinase | 0.42 | 6 | 1 |
| A0A024RDL1 | CCT6A | Chaperonin containing TCP1, subunit 6A (Zeta 1), isoform CRA_a | 0.01 | 2 | 1 |
| A0A087X0X3 | HNRNPM | Heterogeneous nuclear ribonucleoprotein M | 0.01 | 2 | 1 |
| E7EVA0 | MAP4 | Microtubule-associated protein | 0.01 | 1 | 1 |
| A8K486 | PPIA | Peptidyl-prolyl cis-trans isomerase | 0.01 | 8 | 1 |
| A1L0T0 | ILVBL | Acetolactate synthase-like protein | 0.027 | 3 | 1 |
