# Supplementary material for: Clinical Proteomics Profiling for Biomarker Identification Among Patients Suffering With Indian Post Kala Azar Dermal Leishmaniasis
Source: Front Cell Infect Microbiol. 2020 May 27;10:251. doi: 10.3389/fcimb.2020.00251 (PMC7266879; doi:10.3389/fcimb.2020.00251)
Supplement: Supplementary file 3 [file Table_3.DOCX]

**Table S3.** List of up regulated proteins in MAC vs POLY individuals

| **Accession number** | **Gene symbol** | **Approved name** | **Fold change (Mac/Poly)** | **Coverage** | **No. of peptides** |
| --- | --- | --- | --- | --- | --- |
| Q53H26 | TF | Transferrin variant | 25.357 | 30 | 18 |
| P01023 | A2M | Alpha-2-macroglobulin | 66.171 | 16 | 17 |
| C0JYY2 | APOB | Apolipoprotein B | 2.128 | 2 | 10 |
| A0A0G2JPR0 | C4A | Complement C4-A | 100 | 4 | 5 |
| P00734 | F2 | Prothrombin | 100 | 5 | 2 |
| Q7Z3M3 | DKFZp686L04275 | Tubulin alpha chain | 100 | 4 | 1 |
| P10809 | HSPD1 | 60 kDa heat shock protein, mitochondrial | 3.444 | 8 | 3 |
| B2R7F8 | PLG | Plasminogen | 10.62 | 3 | 3 |
| Q99536 | VAT1 | Synaptic vesicle membrane protein VAT-1 homolog | 100 | 7 | 1 |
| Q14240 | EIF4A2 | Eukaryotic initiation factor 4A-II | 1.846 | 5 | 1 |
| P05546 | SERPIND1 | Heparin cofactor 2 | 1.037 | 4 | 2 |
| J3KPS3 | ALDOA | Fructose-bisphosphate aldolase | 100 | 8 | 1 |
| F4ZW62 | ILF2 | NF45 | 100 | 6 | 1 |
| P26641 | EEF1G | Elongation factor 1-gamma | 100 | 3 | 1 |
| B4DR52 | HIST2H2BF | Histone H2B | 4.93 | 5 | 1 |
| P17987 | TCP1 | T-complex protein 1 subunit alpha | 100 | 4 | 1 |
| I3L3D5 | PFN1 | Profilin | 100 | 8 | 1 |
| P22792 | CPN2 | Carboxypeptidase N subunit 2 | 100 | 1 | 1 |
