# Supplementary material for: Clinical Proteomics Profiling for Biomarker Identification Among Patients Suffering With Indian Post Kala Azar Dermal Leishmaniasis
Source: Front Cell Infect Microbiol. 2020 May 27;10:251. doi: 10.3389/fcimb.2020.00251 (PMC7266879; doi:10.3389/fcimb.2020.00251)
Supplement: Supplementary file 4 [file Table_4.DOCX]

**Table S4.** List of down regulated proteins in MAC vs POLY individuals

| **Accession number** | **Gene symbol** | **Approved name** | **Fold change (Mac/Poly)** | **Coverage** | **No. of peptides** |
| --- | --- | --- | --- | --- | --- |
| H6VRG1 | KRT1 | Keratin 1 | 0.253 | 20 | 10 |
| P04003 | C4BPA | C4b-binding protein alpha chain | 0.01 | 11 | 5 |
| P00738 | HP | Haptoglobin | 0.502 | 14 | 5 |
| P02533 | KRT14 | Keratin, type I cytoskeletal 14 | 0.01 | 9 | 3 |
| P13645 | KRT10 | Keratin, type I cytoskeletal 10 | 0.435 | 11 | 6 |
| P35908 | KRT2 | Keratin, type II cytoskeletal 2 epidermal | 0.135 | 8 | 5 |
| P02751 | FN1 | Fibronectin | 0.01 | 2 | 3 |
| P35527 | KRT9 | Keratin, type I cytoskeletal 9 | 0.01 | 10 | 3 |
| P63261 | ACTG1 | Actin, cytoplasmic 2 | 0.01 | 12 | 2 |
| P14625 | HSP90B1 | Endoplasmin | 0.026 | 2 | 1 |
| P25311 | AZGP1 | Zinc-alpha-2-glycoprotein | 0.107 | 11 | 3 |
| P10909 | CLU | Clusterin | 0.01 | 6 | 2 |
| P02671 | FGA | Fibrinogen alpha chain | 0.01 | 1 | 1 |
| P02760 | AMBP | Protein AMBP | 0.01 | 12 | 3 |
| A0A2R8Y6G6 | ENO1 | Alpha-enolase | 0.2 | 8 | 2 |
| P43652 | AFM | Afamin | 0.01 | 4 | 2 |
| B4E1Z4 |  | cDNA FLJ55673 | 0.01 | 4 | 4 |
| Q04695 | KRT17 | Keratin, type I cytoskeletal 17 | 0.578 | 7 | 2 |
| A0A087WT59 | TTR | Transthyretin | 0.01 | 7 | 1 |
| P61513 | RPL37A | 60S ribosomal protein L37a | 0.01 | 20 | 1 |
| P01031 | C5 | Complement C5 | 0.01 | 1 | 2 |
| P02746 | C1QB | Complement C1q subcomponent subunit B | 0.01 | 6 | 1 |
| P04406 | GAPDH | Glyceraldehyde-3-phosphate dehydrogenase | 0.01 | 4 | 1 |
| P27169 | PON1 | Serum paraoxonase/arylesterase 1 | 0.01 | 4 | 1 |
| E9PGN7 | SERPING1 | Plasma protease C1 inhibitor | 0.01 | 3 | 2 |
| P47929 | LGALS7 | Galectin-7 | 0.147 | 10 | 1 |
| C9JC84 | FGG | Fibrinogen gamma chain | 0.01 | 4 | 2 |
| P19827 | ITIH1 | Inter-alpha-trypsin inhibitor heavy chain H1 | 0.01 | 1 | 1 |
| A0A1B1CYC5 | Gc | Vitamin D binding protein (Fragment) | 0.01 | 32 | 1 |
| D6RAR4 | HGFAC | Hepatocyte growth factor activator | 0.477 | 3 | 1 |
| P07900 | HSP90AA1 | Heat shock protein HSP 90-alpha | 0.01 | 3 | 1 |
| C8C504 | HBB | Beta-globin | 0.01 | 8 | 1 |
| P21333 | FLNA | Filamin-A | 0.01 | 1 | 1 |
| A0A0U4BW16 | MYH9 | Non-muscle myosin heavy chain 9 | 0.287 | 1 | 1 |
| P02763 | ORM1 | Alpha-1-acid glycoprotein 1 | 0.01 | 4 | 1 |
| P02790 | HPX | Hemopexin | 0.01 | 2 | 1 |
| C9JF17 | APOD | Apolipoprotein D (Fragment) | 0.01 | 6 | 1 |
| V9GYM3 | APOA2 | Apolipoprotein A-II | 0.01 | 7 | 1 |
| P16402 | HIST1H1D | Histone H1.3 | 0.01 | 5 | 1 |
| O00154 | ACOT7 | Cytosolic acyl coenzyme A thioester hydrolase | 0.01 | 3 | 1 |
| P15822 | HIVEP1 | Zinc finger protein 40 | 0.01 | 1 | 1 |
| D9ZGG2 | VTN | Vitronectin | 0.17 | 4 | 2 |
