# Supplementary material for: Clinical Proteomics Profiling for Biomarker Identification Among Patients Suffering With Indian Post Kala Azar Dermal Leishmaniasis
Source: Front Cell Infect Microbiol. 2020 May 27;10:251. doi: 10.3389/fcimb.2020.00251 (PMC7266879; doi:10.3389/fcimb.2020.00251)
Supplement: Supplementary file 5 [file Table_5.DOCX]

**Table S5.** List of up regulated proteins in MAC vs CR individuals

| **Accession number** | **Gene symbol** | **Approved name** | **Fold change (Mac/CR)** | **Coverage** | **No. of peptides** |
| --- | --- | --- | --- | --- | --- |
| P47929 | LGALS7 | Galectin-7 | 100 | 26 | 2 |
| P07737 | PFN1 | Profilin-1 | 100 | 24 | 2 |
| P61513 | RPL37A | 60S ribosomal protein L37a | 100 | 20 | 1 |
| P02533 | KRT14 | Keratin, type I cytoskeletal 14 | 100 | 19 | 6 |
| P04406 | GAPDH | Glyceraldehyde-3-phosphate dehydrogenase | 100 | 18 | 2 |
| P63261 | ACTG1 | Actin, cytoplasmic 2 | 100 | 18 | 3 |
| P29034 | S100A2 | Protein S100-A2 | 100 | 17 | 1 |
| H6VRG1 | KRT1 | Keratin 1 | 1.293 | 15 | 6 |
| J3KPS3 | ALDOA | Fructose-bisphosphate aldolase | 100 | 14 | 2 |
| P09382 | LGALS1 | Galectin-1 | 100 | 13 | 1 |
| P62913 | RPL11 | 60S ribosomal protein L11 | 100 | 13 | 1 |
| P62805 | HIST1H4A | Histone H4 | 100 | 13 | 1 |
| A0A2R8Y6G6 | ENO1 | Alpha-enolase | 10.117 | 12 | 4 |
| P68363 | TUBA1B | Tubulin alpha-1B chain | 100 | 12 | 3 |
| Q9HCY8 | S100A14 | Protein S100-A14 | 100 | 11 | 1 |
| P01023 | A2M | Alpha-2-macroglobulin | 17.516 | 10 | 9 |
| D3DSW3 | PROSC | Proline synthetase co-transcribed homolog | 100 | 10 | 1 |
| G8JLB6 | HNRNPH1 | Heterogeneous nuclear ribonucleoprotein H | 100 | 10 | 2 |
| P01591 | JCHAIN | Immunoglobulin J chain | 100 | 9 | 1 |
| B3KVF5 | KRT15 | cDNA FLJ16494 fis | 12.576 | 9 | 2 |
| P08779 | KRT16 | Keratin, type I cytoskeletal 16 | 100 | 9 | 3 |
| A0A024R7M0 | TMED9 | Transmembrane emp24 protein transport domain containing 9 | 100 | 9 | 1 |
| I3L0A0 | TMEM189-UBE2V1 | HCG2044781 | 100 | 8 | 1 |
| A0A109NGN6 | PSMA5 | Proteasome subunit alpha type | 100 | 8 | 1 |
| A0A024R7T3 | HNRPF | Heterogeneous nuclear ribonucleoprotein F | 100 | 7 | 1 |
| Q04695 | KRT17 | Keratin, type I cytoskeletal 17 | 100 | 7 | 2 |
| O43866 | CD5L | CD5 antigen-like | 100 | 7 | 1 |
| Q53HV2 | CCT7 | Chaperonin containing TCP1 | 100 | 7 | 2 |
| G3V5Z7 | PSMA6 | Proteasome subunit alpha type | 11.032 | 7 | 1 |
| Q99536 | VAT1 | Synaptic vesicle membrane protein VAT-1 homolog | 100 | 7 | 1 |
| F4ZW62 | ILF2 | NF45 | 100 | 6 | 1 |
| P60174 | TPI1 | Triosephosphate isomerase | 100 | 6 | 1 |
| A8K237 | CYB5R2 | NADH-cytochrome b5 reductase | 100 | 6 | 1 |
| P68371 | TUBB4B | Tubulin beta-4B chain | 7.375 | 6 | 1 |
| P15880 | RPS2 | 40S ribosomal protein S2 | 100 | 6 | 1 |
| P05155 | SERPING1 | Plasma protease C1 inhibitor | 100 | 6 | 2 |
| P00558 | PGK1 | Phosphoglycerate kinase 1 | 100 | 6 | 1 |
| A0A2U3TZH3 | EEF1A2 | Elongation factor 1-alpha 2 | 100 | 5 | 2 |
| A8K455 | MAT1A | S-adenosylmethionine synthase | 2.651 | 5 | 1 |
| P08238 | HSP90AB1 | Heat shock protein HSP 90-beta | 100 | 5 | 2 |
| Q14240 | EIF4A2 | Eukaryotic initiation factor 4A-II | 100 | 5 | 1 |
| P13010 | XRCC5 | X-ray repair cross-complementing protein 5 | 100 | 5 | 1 |
| P02763 | ORM1 | Alpha-1-acid glycoprotein 1 | 100 | 4 | 1 |
| Q16658 | FSCN1 | Fascin | 100 | 4 | 2 |
| P07900 | HSP90AA1 | Heat shock protein HSP 90-alpha | 100 | 4 | 2 |
| B2R9U2 | FKBP4 | Peptidylprolyl isomerase | 100 | 4 | 1 |
| Q9UNS2 | COPS3 | COP9 signalosome complex subunit 3 | 100 | 4 | 1 |
| A0A024RAC5 | RCC2 | Regulator of chromosome condensation 2 | 100 | 4 | 1 |
| B4DY90 | TUBB | Tubulin beta chain | 100 | 4 | 1 |
| Q86U75 |  | Dihydropyrimidinase-like 2 | 100 | 4 | 1 |
| A1L0T0 | ILVBL | Acetolactate synthase-like protein | 100 | 3 | 1 |
| A0A024R5Z9 | PKM2 | Pyruvate kinase | 100 | 3 | 1 |
| P26641 | EEF1G | Elongation factor 1-gamma | 6.084 | 3 | 1 |
| B2R7F8 | PLG | Plasminogen | 1.06 | 3 | 2 |
| D6RAR4 | HGFAC | Hepatocyte growth factor activator | 100 | 3 | 1 |
| P19338 | NCL | Nucleolin | 100 | 3 | 1 |
| P14625 | HSP90B1 | Endoplasmin | 100 | 2 | 1 |
| D6REX3 | SEC31A | Protein transport protein Sec31A | 100 | 2 | 1 |
| P05556 | ITGB1 | Integrin beta-1 | 100 | 2 | 1 |
| P00734 | F2 | Prothrombin | 74.995 | 2 | 1 |
| P12814 | ACTN1 | Alpha-actinin-1 | 1.633 | 2 | 1 |
| Q86UP2 | KTN1 | Kinectin | 100 | 2 | 1 |
| P05546 | SERPIND1 | Heparin cofactor 2 | 100 | 2 | 1 |
| A0AVT1 | UBA6 | Ubiquitin-like modifier-activating enzyme 6 | 100 | 2 | 1 |
| P49327 | FASN | Fatty acid synthase | 7.502 | 2 | 2 |
| A4QPB0 | IQGAP1 | IQ motif containing GTPase activating protein 1 | 100 | 1 | 1 |
| A0A0U4BW16 | MYH9 | Non-muscle myosin heavy chain 9 | 100 | 1 | 1 |
| P21333 | FLNA | Filamin-A | 100 | 1 | 2 |
| J7M2B1 | EZR-ROS1 | Tyrosine-protein kinase receptor | 100 | 1 | 1 |
| A0A0D9SGF6 | SPTAN1 | Spectrin alpha chain, non-erythrocytic 1 | 100 | 1 | 1 |
| A0A024R321 | FLNB | Filamin B, beta | 100 | 1 | 1 |
