# Supplementary material for: Clinical Proteomics Profiling for Biomarker Identification Among Patients Suffering With Indian Post Kala Azar Dermal Leishmaniasis
Source: Front Cell Infect Microbiol. 2020 May 27;10:251. doi: 10.3389/fcimb.2020.00251 (PMC7266879; doi:10.3389/fcimb.2020.00251)
Supplement: Supplementary file 6 [file Table_6.DOCX]

**Table S6.** List of down regulated proteins in MAC vs CR individuals

| **Accession number** | **Gene symbol** | **Approved name** | **Fold change (Mac/CR)** | **Coverage** | **No. of peptides** |
| --- | --- | --- | --- | --- | --- |
| P02787 | TF | Serotransferrin | 0.01 | 23 | 13 |
| C9JC84 | FGG | Fibrinogen gamma chain | 0.019 | 18 | 4 |
| P02749 | APOH | Beta-2-glycoprotein 1 | 0.085 | 17 | 3 |
| D6RF35 | GC | Vitamin D-binding protein | 0.03 | 16 | 5 |
| B0YIW2 | APOC3 | Apolipoprotein C-III | 0.01 | 14 | 1 |
| B4E1C2 | KNG1 | Kininogen 1, isoform CRA_b | 0.052 | 11 | 5 |
| P35908 | KRT2 | Keratin, type II cytoskeletal 2 epidermal | 0.247 | 11 | 4 |
| A0A024R3E3 | APOA1 | Apolipoprotein A-I, isoform CRA_a | 0.011 | 7 | 2 |
| P02790 | HPX | Hemopexin | 0.01 | 7 | 2 |
| V9GYM3 | APOA2 | Apolipoprotein A-II | 0.01 | 7 | 1 |
| P04003 | C4BPA | C4b-binding protein alpha chain | 0.033 | 7 | 2 |
| A8K4W0 | RPS3A | 40S ribosomal protein S3a | 0.058 | 5 | 1 |
| P17987 | TCP1 | T-complex protein 1 subunit alpha | 0.05 | 4 | 1 |
| P43652 | AFM | Afamin | 0.01 | 2 | 1 |
| P02751 | FN1 | Fibronectin | 0.01 | 2 | 2 |
| P35527 | KRT9 | Keratin, type I cytoskeletal 9 | 0.01 | 1 | 1 |
| P10909 | CLU | Clusterin | 0.01 | 1 | 1 |
| P01031 | C5 | Complement C5 | 0.01 | 1 | 1 |
| C0JYY2 | APOB | Apolipoprotein B (Including Ag(X) antigen) | 0.01 | 1 | 2 |
